# Development of Polymeric Micelles for Combined Delivery of Luteolin and Doxorubicin for Cancer Therapy

**DOI:** 10.7150/jca.96402

**Published:** 2024-07-08

**Authors:** Saad Alshamrani, Ashok Kumar, Mohammed S. Aldughaim, Khalid M. Alghamdi, Muhammad Delwar Hussain, Fars K. Alanazi, Mohsin Kazi

**Affiliations:** 1 Kayyali Chair for Pharmaceutical Industries, Department of Pharmaceutics, College of Pharmacy, King Saud University, Riyadh 11451, Kingdom of Saudi Arabia.; 2 Department of Pharmaceutics, College of Pharmacy, King Saud University, Riyadh 11451, Kingdom of Saudi Arabia.; 3 Vitiligo Research Chair, Department of Dermatology, College of Medicine, King Saud University, Riyadh, Kingdom of Saudi Arabia.; 4 Research Center, King Fahad Medical City, Riyadh Second Health Cluster, Riyadh, 11525, Kingdom of Saudi Arabia.; 5 Department of Dermatology, College of Medicine, King Saud University, Riyadh, Kingdom of Saudi Arabia.; 6 Department of Pharmaceutical Sciences, School of Pharmacy and Health Professions, University of Maryland Eastern Shore, Princess Anne, MD 21853, USA.

**Keywords:** Doxorubicin, luteolin, polymeric micelles, cancer, combination therapy

## Abstract

**Background:** Luteolin (LUT) is a bioactive compound with several pharmacological activities including anticancer effect. Doxorubicin (DOX) is an anthracycline chemotherapeutic drug that have proven to be effective in treating various types of cancers. Polymeric micelles (PMs) containing biologically active materials have emerged as prospective dosage forms with high drug-loading, which can add therapeutic benefit to the poorly water-soluble compounds and novel chemical entities. PMs are effective in delivering several drugs, such as anticancer drugs, antifungal drugs, flavonoids and drugs targeting the brain. The aim of the current study is to develop PMs for LUT and DOX as a combined delivery system for cancer therapy.

**Methods:** PMs were prepared using 2.5% of each of LUT and DOX with varying compositions of Poloxamer 188, Poloxamer 407, Vitamin E (TPGS), Poloxamer 123 and Gellucire 44/14 at room temperature. Particle size, polydispersity index, zeta potential, were achieved using Zetasizer Nano particle size analyzer and the sizes were further confirmed with transmission electron microscopy (TEM). Prepared PMs were further characterized using powder X-ray diffraction (PXRD) and fourier transform infrared spectroscopy (FTIR). An MTT assay was performed on breast cancer (MCF-7) cells and liver cancer (HepG2) cells to determine the cytotoxic effect of the different PMs formulations.

**Results:** PMs were successfully developed and optimized using 74.3% Poloxamer 407 with 20.7% Vitamin E (TPGS), and 70% Poloxamer 407 with 25% Gellucire 44/14, respectively. The droplet size and polydispersity index were found to be 62.03 ± 3.99 nm, 91.96 ± 5.80 nm and 0.33 ± 0.05, 0.59± 0.03, respectively for PMs containing TPGS and Gellucire 44/14. Zeta potentials of the PMs containing TPGS and Gellucire 44/14 were recorded as -2.27 ±0.11mV and -7.78 ± 0.10 mV, respectively. The PMs showed a spherical structure with approximately 50-90 nm range evident by TEM analysis. The PXRD spectra of PMs powder presented the amorphization of LUT and DOX. The FTIR spectra of LUT-loaded and DOX-loaded PMs were identical, suggesting consistent PMs composition. The MTT assay showed that the representative combined drug loaded PMs treatment led to a reduction in the viability of MCF-7 and HepG2 cells compared to drug free PMs and pure LUT, DOX alone.

**Conclusions:** PMs with LUT and DOX exhibited significant cytotoxic effects against breast and liver cancer cells and could thus be an important new pharmaceutical formulation to treat cancer.

## 1. Introduction

Luteolin (LUT) is a polyphenolic compound that can be found in various fruits and vegetables, such as pomegranate, cabbage, celery, and spinach. The mean estimated daily dietary intake of LUT supplementation is approximately 0.1 mg 1. LUT has pharmacological effects such as anticancer, anti-inflammatory, neuroprotective, and cardioprotective effects 2-4. LUT activates apoptotic cell death by triggering apoptosis pathways and suppressing cell survival pathways. Furthermore, it can produce anticancer effects by inducing cell cycle arrest, senescence, or apoptosis in different cancer cells. In prostate cancer, LUT can inhibit proliferation and induce apoptosis. LUT also inhibits tumor growth against breast cancer cell lines (MCF-7/6 and MDA-MB-231) 5. Despite its efficacy and low toxicity, the use of LUT is in demand but limited due to its poor water solubility and low bioavailability 6. Different formulations have been used to enhance the solubility and bioavailability of LUT, including phospholipid complexation, cyclosophoraose complexation and loading in polymeric micelles (PMs) 7. Encapsulation of LUT into liposomes and zein nanoparticles also showed enhanced solubility and bioavailability and increased anticancer activity 8, 9.

Doxorubicin (DOX) is an anthracycline chemotherapeutic drug that has proven to be effective in treating various types of cancer. It can be resistant to gastric cancer, acute myeloid leukemia, lung cancer, lymphoma and breast cancer 10. DOX works as a DNA-intercalating agent and a topoisomerase II inhibitor 11. Currently, DOX is only available as an intravenous dosage form. Unfortunately, DOX can lead to serious side effects, including cardiotoxicity, nephrotoxicity and hepatotoxicity 12. For example, to overcome cardiotoxicity after intravenous administration, several novel carriers have been used, such as novel pectin-adriamycin conjugates, N-(2-hydroxypropyl)-loaded poly butylcyanoacrylate nanoparticles, DOX-loaded polymerases, polyisohexylcyanoacrylate nanoparticles, and dextran-DOX/chitosan nanoparticles. Nanotechnology has been effective in delivering DOX and has been proven to increase antineoplastic activity and decrease side effects although passive or active targeting 13. Several drug delivery systems using PMs have been used to improve DOX efficacy and enhance solubility, such as liposomes 14, gold nanoparticles 15, dendrimers 16 and amino acid-modified B-cyclodextrin platinum complexes 17.

Nanocarriers are one of the most promising systems developed to deliver hydrophobic drugs such as LUT and DOX. Polymeric micelles (PMs) are nanoparticles that are widely used to enhance the solubility of poorly water-soluble drugs such as DOX 18. PMs produce nanoparticles (10-100 nm) that are spherical and colloidal and form self-assembled amphiphilic block copolymers that contain hydrophobic cores and hydrophilic shells 19. PMs can target tumors through a passive targeting mechanism called the enhanced permeability and retention (EPR) effect 17,20. PMs showed higher drug loading capacity, tumor-specific uptake and improved anticancer effects along with reduced side effects of drugs 20. These effects were made possible by modifying the PMs shell by attaching specific ligands to encourage PMs-cell specific interactions 21. One of the common effective polymers used in polymeric micelle development is poloxamer 407, a hydrophilic nonionic surfactant that enhances the solubility of hydrophobic drugs and shows better cytotoxic properties 22. Another common polymer micelle is d-α-tocopheryl polyethylene glycol succinate (TPGS), which has an amphiphilic structure and works as a cosurfactant with antioxidant effects that can also improve bioavailability and drug targeting 23. Most interestingly, Gelucire 44/14 is a nonionic surfactant that has been used to improve solubility, dissolution rate and stability in some drugs, such as simvastatin, albendazole and olanzapine 24. In the current study, we aimed to load LUT alone and in combination with DOX using biocompatible polymers as PMs to improve the efficacy of therapy. The polymers TPGS: Poloxamer407 and Gelucire44/14: Poloxamer407 at 7:3 and 5:5 molar ratios, respectively, will be prepared by using the thin-film hydration method, which was explained in detail by Y. Zhang et al. 20. This method involves making a thin lipid film in a round-bottom flask by the removal of organic solvent. The PMs will be lyophilized, characterized and investigated for their effects against breast and liver cancer cells (MCF-7 & HepG2).

## 2. Materials

Luteolin (LUT, purity = 98.5%) was supplied by Enzo life Sciences, (Lausen, Switzerland). Doxorubicin (DOX, purity = 99.8%) was obtained from Sigma Aldrich, (MO, USA). TPGS was purchased from Sigma‒Aldrich (St Louis, MO, USA). Poloxamer 407 (P407) was purchased from BASF Corporation (Florham Park, NJ, USA). Gellucire 44/14 was obtained from Gattefosse Co. Ltd. (Saint-priest, France). High-purity Milli-Q water was obtained through a Milli-Q Integral Water Purification System (Millipore, Bedford, MA). All other chemicals and solvents were of analytical purity or HPLC grade.

### 2.1. Reagents for MTT Assay

All tissue culture media and materials including DMEM, L-glutamine, penicillin/streptomycin, Trypsin-EDTA solution and Fetal Bovine Serum, were obtained from Gibco Inc. (NY, USA). Cell culture flasks (25 and 75 cm^2^) with vent cap, Falcon^®^ 15- and 50-mL polystyrene centrifuge tubes and sterile individually-wrapped StripetteTM serological polystyrene pipettes were purchased from Corning^®^ USA. All protein chemistry reagents and buffers were obtained from Bio-Rad Laboratories GmbH (Munich, Germany).

## 3. Methods

### 3.1. Preparation of Drug Free PMs, LUT-PMs, DOX-PMs and LUT-DOX-PMs

Drug-free PMs (DF-PMs), LUT-loaded (LUT-PMs)/DOX-loaded (DOX-PMs) and LUT & DOX-loaded (LUT-DOX-PMs) micelles were prepared by thin film hydration methods. Briefly, P407 with TPGS and P407 with Gellucire 44/14 at 10 mM concentrations at ratios of 7:3 and 5:5, respectively, with LU alone and in combination with DOX were dissolved in ethanol in a round bottom flask. Then, the round bottom flask was placed into a vacuum chamber for drying ethanol. After overnight of vacuuming, the flask was removed from the vacuum chamber and reconstituted with distilled water. The samples were kept at 4 °C until further characterization studies.

### 3.2. Particle Size, Polydispersity Index (PDI) and Zeta Potential Analysis

The particle size distribution and PDI of LUT-PMs/DOX-PMs and LUT-DOX-PMs were measured utilizing a particle size analyzer. In addition, the zeta potential, which depicts the surface charge of the dispersing formulation, was measured. The samples for both particle size and zeta potential were prepared by diluting the anhydrous formulation with water at a ratio of 1: 1000 *v/v* and mixing for 1 min before testing. Then, the diluted formulations were transferred into cuvettes to analyze each sample. The experiments were performed in triplicate.

### 3.3. Drug Loading and Encapsulation Efficiency

Drug loading (DL%) and encapsulation efficiency (EE%) were determined according to the drug concentrations available in PMs. The drug-loaded PMs were analyzed by UHPLC using UV‒Vis spectrophotometry at maximum absorbances (λmax) of 495 nm for DOX and 333 nm for LUT. The lyophilized PMs were appropriately diluted with acetonitrile prior to analysis.

DL% and EE% were calculated using the following equations:

DL% = (Weight of the drug in micelles/Weight of the feeding polymer and drug) × 100

EE% = (Weight of the drug in micelles/Weight of the feeding drug) × 100

### 3.4. Lyophilization of PMs

Vials with the PM dispersions were frozen in an ultracold refrigerator at -80 °C for ~2 hrs at a tilted angle (~30 degrees). The frozen PM dispersion was transferred into a lyophilizer and lyophilized to obtain a dry fluffy powder. Lyophilized polymeric micelles were kept in amber glass scintillation vials, tightly closed at -80 °C until use.

### 3.5. Transmission Electron Microscopy (TEM) Analysis

TEM was used to analyze the morphology of DF-PMs, LUT-PMs/DOX-PMs and LUT-DOX-PMs. A drop of the diluted sample (with water) was placed on a 300-mesh carbon-coated copper grid. Excess liquid was removed using filter paper, and the grid was left to air dry. Then, a drop of 1% phosphotungstic acid in water was added to the grid, left for 5 mins to settle down and dried as previously described. Finally, the dried grid was visualized at an operating voltage of 80 kV, and the images of PMs were captured 25.

### 3.6. Fourier transform infrared spectroscopy (FTIR) Analysis

Fourier transform infrared (FTIR) studies were conducted to investigate potential interactions between the drugs LUT and DOX and polymeric micelles. The chemical properties and complexation of powdered samples were analyzed using an FTIR spectrometer (specifically, the FTIR Spectrum BX from Perkin Elmer LLC, USA) 26. Pure LUT, pure DOX, and PMs powders were compressed for 5 mins at 5 bars using a KBr press. The resulting spectra were scanned over the wavenumber range of 400-4,000 cm⁻¹.

### 3.7. X-Ray Diffraction (XRD) Analysis

Powder crystallinity was assessed by a multipurpose X-ray diffractometer. DF-PMs, LUT-PMs/DOX-PMs and LUT-DOX-PMs samples were analyzed using CuKα radiation of wavelength 1.54056 Å, generated at 40 kV voltage, 40 mA current and receiving slit of 0.3 mm. Analyses were performed over a 2θ range of 3-60° with an angular increment of 0.5°/min and scan step time of 1.0 sec 25.

### 3.8. *In Vitro* Drug Release Studies

The *in vitro* drug release behavior from LUT and DOX was monitored in simulated body fluid (SBF) (pH 7.4) with 0.5% Tween-80. SBF has an ionic concentration similar to human plasma, and Tween-80 were added to the release media to maintain the sink condition. Briefly, 10 mg of LUT-PMs and LUT-DOX-PMs were introduced into a dialysis membrane bag, and the sealed dialysis bag was incubated in 20 mL of release media in an orbital shaker at 37 °C with 100 rpm agitation. At predetermined time intervals, samples were withdrawn and replaced with fresh release media. The concentrations of LUT and DOX in a sample was measured using the UHPLC analysis method at maximum spectrophotometric absorbances of 495 nm for DOX and 333 nm for LUT, and the cumulative release percentage were calculated.

### 3.9. Cell Viability Measurement of DF-PMs, LUT-PMs/DOX-PMs and LUT-DOX-PMs

Cell viability experiments were conducted using 3-[4,5dimethylthiazol-2-yl]-2,5-diphenyltetrazolium bromide (MTT) assay method with breast (MCF-7) and liver (HepG2) cancer cell lines. Cancer cells were treated with various concentrations such as 0.78, 1.56, 3.12, 6.25, 12.5, 25, 50 and 100 µg/mL, of DF-PMs, LUT-PMs/DOX-PMs and LUT-DOX-PMs. The cytotoxic effect of the DF-PMs, LUT-PMs/DOX-PMs and LUT-DOX-PMs were evaluated by testing the capacity of the reducing enzymes present in viable cells to convert MTT to formazan crystals. After 72 hrs of incubation with different concentrations of above mentioned drugs, the media were discarded, and adherent cells were incubated with 100 µL/well MTT at a concentration of 0.5 mg/mL prepared in PBS and subsequently incubated at 37ºC for additional 3 hrs at 37^o^C under dark condition 24. Then, 100 µL isopropyl alcohol was added per well to dissolve the purple formazan crystals with the help of shaking for another 2 hrs at room temperature. Subsequently, the absorbance was measured at 549 nm using ELX 800 BioTek microplate reader (BioTek Instruments, Winooski, VT, USA). The results were analyzed in triplicates and the viability percentage was calculated. The cytotoxicity of different formulations was determined by testing the capacity of the reducing enzymes present in the viable cells to convert MTT to formazan crystals 25. Concentrations causing 50% inhibition of growth (IC_50_) of MCF-7 breast cancer cells and HepG2 liver cancer cells were calculated by use of Microsoft Excel trendline equation 24.

### 3.10. UHPLC Analysis

The amounts of LUT and DOX in the investigated samples were quantified using an UHPLC method by injecting a 5 µL sample into the UHPLC systems. Phosphate buffer was used as a mobile phase (40 mM phosphate buffer adjusted to pH 7.0 using 10% *w/v* potassium hydroxide) and was pumped through a C18 column (2.1 × 4.6) at a rate of 0.4 mL/min. The UV‒Vis detector was set at 495 nm to detect DOX and 333 nm for LUT in each sample. All operations were carried out at room temperature.

### 3.11. Statistical Analysis

The data were expressed as the mean ± SD and analyzed statistically (Graph Pad Prism, version 4.5) using one-way analysis of variance (ANOVA) and considered statistically significant when *p* <0.05.

## 4. Results and Discussion

### 4.1. Formulation Development of Polymeric Micelles (PMs)

PMs have emerged as multifunctional nanoparticles with promise in various scientific fields, including cancer therapy 27. Biocompatible and bio-related copolymers play a crucial role in drug delivery, enhancing treatment efficacy and minimizing toxicity. The PMs used in this study were developed successfully using five different polymers for the combined LUT and DOX delivery systems. A total 5% drug loading was present either alone or in combination with two drugs in all batches except batch 5 (B5). Poloxamer 407 was used in all the Formulations (B1-B6). Drug-free formulations (BL1 and BL2) were developed using P407 and TPGS or GC. B1 and B2 formulations contained 74.3% P407 and 20.7% TPGS with 5% LUT and 70.0% P407 and 25% GC with 5% DOX, respectively (Table 1). Similarly, B3 and B4 formulations contained 74.3% P407 and 20.7% TPGS with 2.5% LUT & 2.5% DOX and 70.0% P407 and 25% GC with 2.5% LUT & 2.5% DOX, respectively. Formulation B5 was produced with 9.45% P407 and P188 73.5% with 12.6% LUT. On the other hand, B6 was produced with 31.9% P407 and PDLG 63.1% with 5% LUT.

### 4.2. Particle Size, Polydispersity Index (PDI) and Zeta Potential Before Lyophilisation

Particle size was measured for six different formulations using a particle size analyzer. Based on the molar ratio, the concentrations of the formulations were B1 (74.3% P407-20.7% TPGS with 5% LUT), B2 (70% P407-25% GC with 5% DOX), B3 (74.3% P407-20.6% TPGS with 2.5% LUT with 2.5% DOX), B4 (70% P407-25% GC with 2.5% LUT with 2.5% DOX), B5 (73.5% P188-9.45% P407 with 12.6% LUT) and B6 (63.08% PDLG-31.9% P407 with 5% LUT). B5 and B6 showed large particle sizes. B1 and B2 have larger sizes than B3 and B4, which could be due to loading 5% of LUT. The B1, B2, B3, B4 and BL1 formulations displayed nanoscale sizes and were chosen for further investigations (Table 2). The particle size analysis showed that formulations B5 and B6 exhibited larger particle sizes compared to the others, suggesting potential challenges in terms of stability or bioavailability. These findings underscore the importance of considering particle size in formulation development to optimize stability and therapeutic efficacy.

### 4.3. Drug Solubility and Loading of Polymeric Micelles

LUT and DOX solubility and their percent loading are shown in Table 3. The data from Table 3 depicts that LUT has a higher loading capacity than DOX in the combined dosage formulations. The observed differences in % drug load may be attributed to the complex interactions between LUT, DOX, and the components of the Polymeric Micelles (PMs) during the preparation and lyophilization process, which could influence the encapsulation efficiency and drug-loading capacity of the PMs. Additionally, B4 contains GC (consisting of a small fraction of mono-, di-, and triglycerides, and mainly PEG-32 mono- and diesters of lauric acid) which, when combined in lipid-polymeric micelles, may lead to improved solubility for luteolin and doxorubicin due to the hydrophobic environment provided by the micelle core.

### 4.4. Particle Size, PDI and Zeta Potential Value of PMs After Lyophilization

Table 4 shows the particle size, polydispersity index (PDI), and zeta potential values of four batches of polymeric micelles after lyophilization. Polymeric micelles are nanosized aggregates of amphiphilic copolymers that can be used for drug delivery 28. Lyophilization is a freeze-drying process that can improve the stability and storage of polymeric micelles, but it may also affect their properties and performance. Therefore, it is important to measure the parameters that indicate the size, uniformity, and stability of the micelles after lyophilization, such as particle size, PDI, and zeta potential 29.

Particle size affects the drug loading capacity, pharmacokinetics, biodistribution, and cellular uptake of the micelles. Smaller micelles are generally preferred, as they can avoid rapid clearance by the reticuloendothelial system and enhance the permeability and retention effect in tumor tissues. Findings here showed that the particle size of the micelles increased for micelles that were loaded with 2.5% each of both LUT and DOX compared to the micelles containing 5% of either drug alone. This may be due to the different drugs loaded in the micelles, as previous studies have shown that DOX and quercetin, a flavonoid similar is structure and biological function to quercetin have different molecular weights, solubilities, and interactions with the copolymer 30.

PDI is a measure of the size distribution of the micelles, ranging from 0 to 1. It indicates the uniformity and homogeneity of the micelles, which are important for consistent drug delivery and reduced toxicity. Lower PDI values are desirable, as they imply narrower size distribution and less aggregation 31. The table shows that the PDI values of the micelles are relatively low, except for B4, which has a high PDI of 0.793333. This suggests that B4 has a wide range of micelle sizes and may be prone to aggregation and instability. The PDI values of B2 and B3 are similar and lower than B1, indicating that they have more uniform micelles. The PDI values may be influenced by the lyophilization process, the cryoprotectant used, and the drug loading efficiency 31.

Zeta potential is the electric potential at the surface of the micelles, measured in millivolts (mV). It reflects the electrostatic repulsion between the micelles, which affects their stability and aggregation tendency. Higher absolute values of zeta potential indicate stronger repulsion and higher stability, while lower values indicate weaker repulsion and higher aggregation risk. The zeta potential values vary among the batches, with B3 having the highest value (-2.76 mV) and B4 having the lowest value (-7.97667 mV). This implies that B4 has the most stable micelles, while B3 has the least stable micelles. The zeta potential values may be affected by the pH of the solution, the drug loading, and the lyophilization conditions².

### 4.5. Transmission Electron Microscopy Analysis

**Figure 1:**
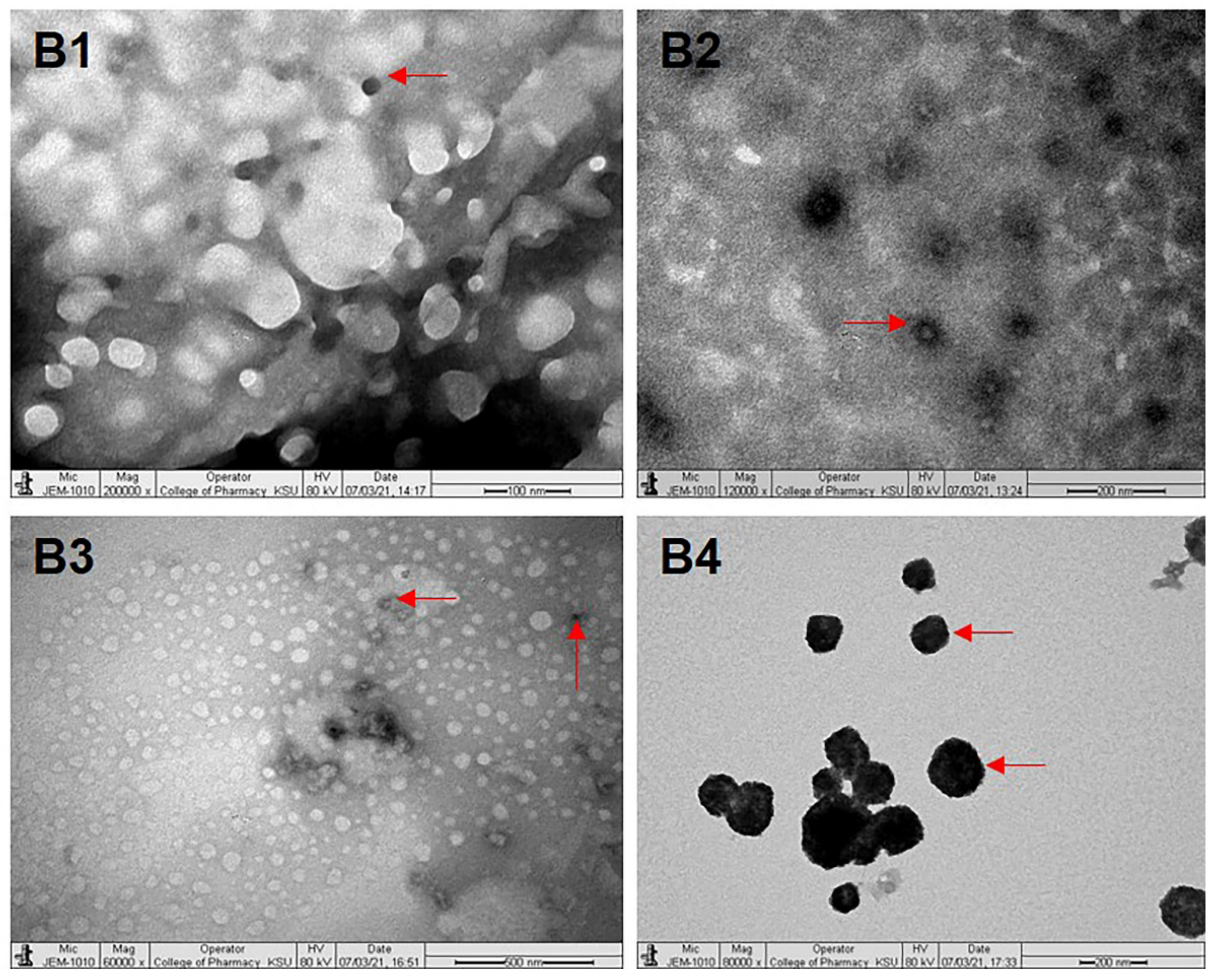
Images of B1 (a), B2 (b), B3 (c) and B4 (d) under transmission electron microscopy. B1, B2, B3 and B4 represent LUT-PMs, DOX-PMs, LUT-DOX-PMs with TPGS and LUT-DOX-PMs with GC, respectively. The red arrows indicate the size and shape of PMs particles in different batches.

Under TEM, B1 (P407-TPGS/LUT) showed a spherical and uneven distribution similar to that expected by droplet sizing (Fig. 1B1). B2 (P407-GC/DOX) showed a spherical and uniform distribution under TEM image (Fig. 1B2). Under TEM, B3 (P407-TPGS/LUT+DOX) displayed a spherical and uniform distribution (Fig. 1B3). Also, B4 (P407-GC/LUT+DOX) displayed a spherical and uniform distribution under TEM (Fig. 1B4).

### 4.6. X-Ray Diffraction (XRD) Analysis

**Figure 2:**
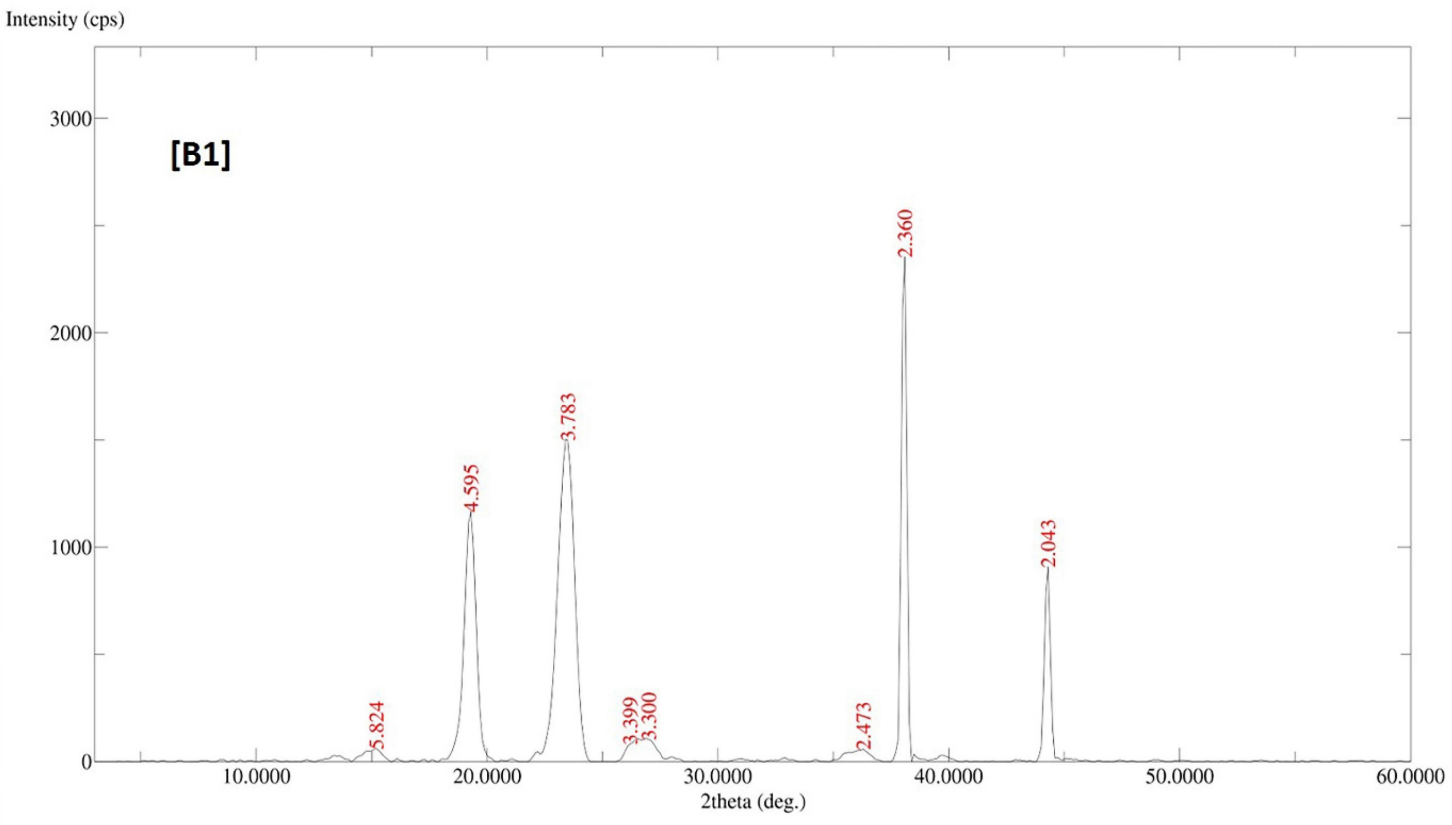

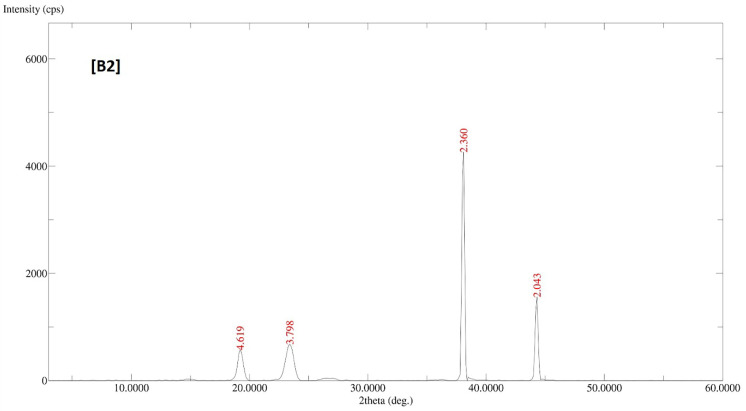

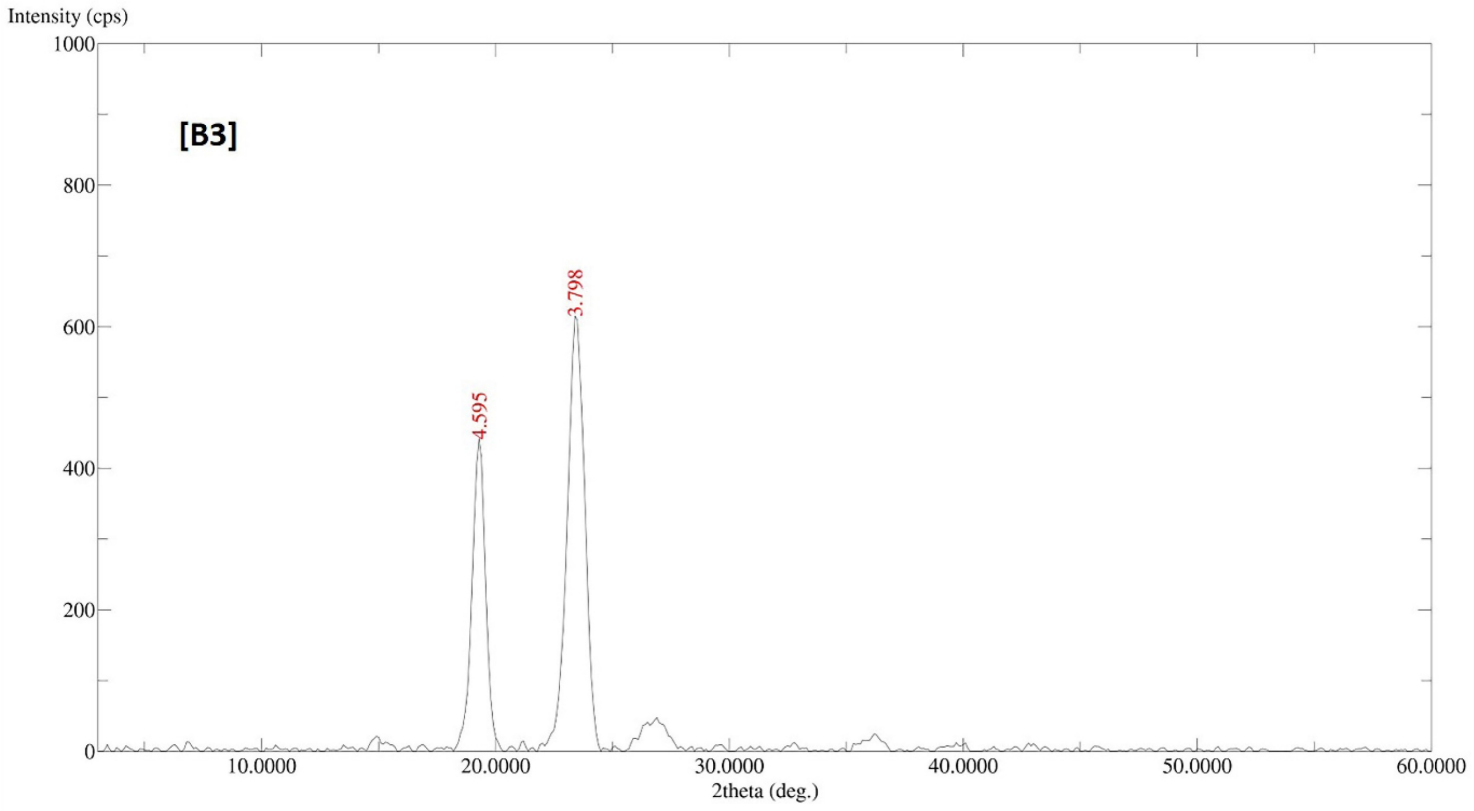

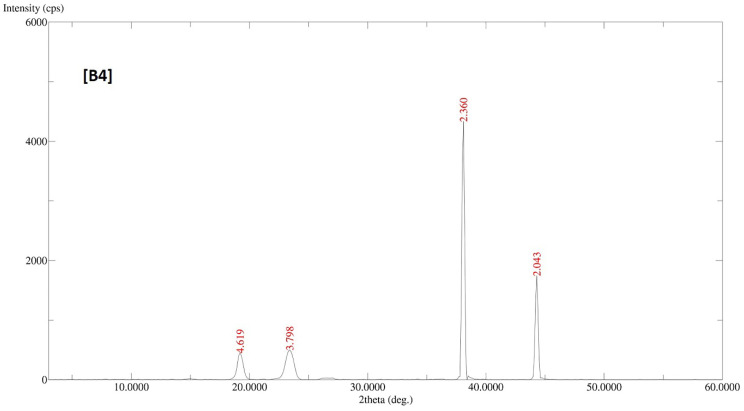
XRD patterns of batches B1, B2, B3 and B4 of polymeric micelles. B1, B2, B3 and B4 represent LUT-PMs, DOX-PMs and LUT-DOX-PMs, respectively.

The diffractogram patterns of all samples were determined using XRD analysis. B1 showed sharp peaks at 5.824, 4.595, 3.783, 3.399, 3.300, 2.473, 2.360 and 2.043, which indicated the crystalline structure of the formulation (Fig. 2A). B2 also showed sharp peaks at 4.619, 3.798, 2.360 and 2.043, which expressed the crystalline structure of the formulation (Fig. 2B). B3 showed two sharp peaks at 4.595 and 3.798, indicating the crystalline state of the formulation (Fig. 2C). B4 showed sharp peaks at 4.619, 3.798, 2.360 and 2.043, displaying the crystalline state of the formulation (Fig. 2D).

### 4.7. FTIR Analysis

**Figure 3:**
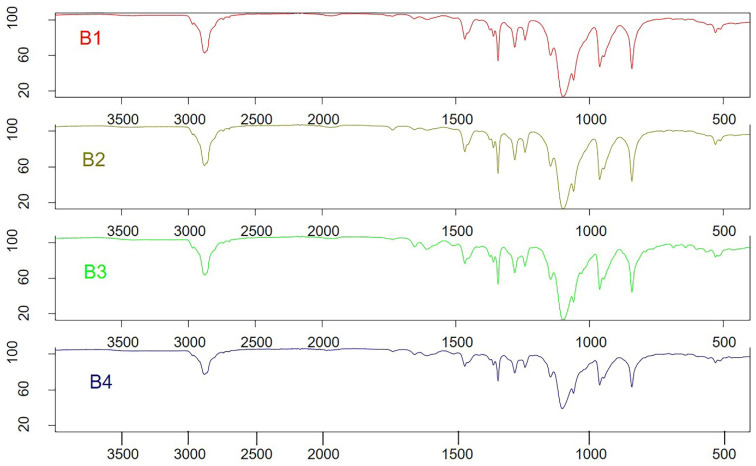
FTIR chromatograms of the B1-B4 batches of polymeric micelles. B1, B2, B3 and B4 represent LUT-PMs, DOX-PMs and LUT-DOX-PMs, respectively.

The LUT-PMs, DOX-PMs, and LUT-DOX-PMs were initially designed as distinct products. Consequently, no interaction between the two drugs was expected. For FTIR analysis, samples were prepared by loading either luteolin (LUT) or doxorubicin (DOX) individually into each PMs (polymeric micelles). Surprisingly, the FTIR spectra of LUT-loaded and DOX-loaded PM formulations were identical, indicating that the PM composition remained consistent (as shown in Figure 3, batch B1 and B2). Furthermore, no significant changes in FTIR spectra were observed between LUT-DOX-PMs formulated with TPGS (d-α-tocopheryl polyethylene glycol 1000 succinate) and those formulated with GC (Gellucire 44/14).

### 4.8. LUT and DOX-PMs Release Studies

**Figure 4:**
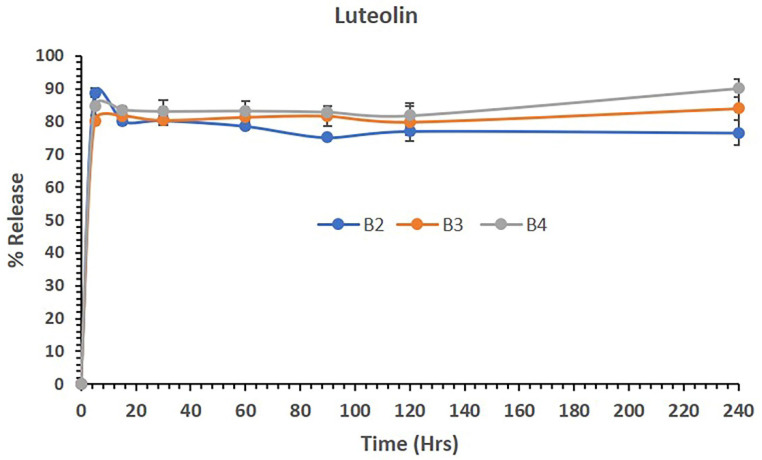
The percent release of LUT from the polymeric micelles of batches B1, B3 and B4 in aqueous media over 240 mins. The data are presented as the mean ±SD (n=3).

Figure 4 shows the release of LUT from polymeric micelles of batches B1, B3 and B4 in aqueous media. The data showed that LUT-loaded polymeric micelles (B1) released 88.83% of the drug in 5 mins and maintained almost 76% of the LUT in solution until 4 hrs (240 mins). Similarly, the combination of LUT and DOX loaded in B3 and B4 PM batches released approximately 80% and 84% of LUT in 5 mins, respectively. The combined dosage forms B3 and B4 continued to be released up to 84% and 90% and maintained the drug in solution until 240 mins. The overall release studies suggest that LUT was released from the combined dosage forms effectively without precipitation, which might be a potential delivery system for active therapy.

**Figure 5:**
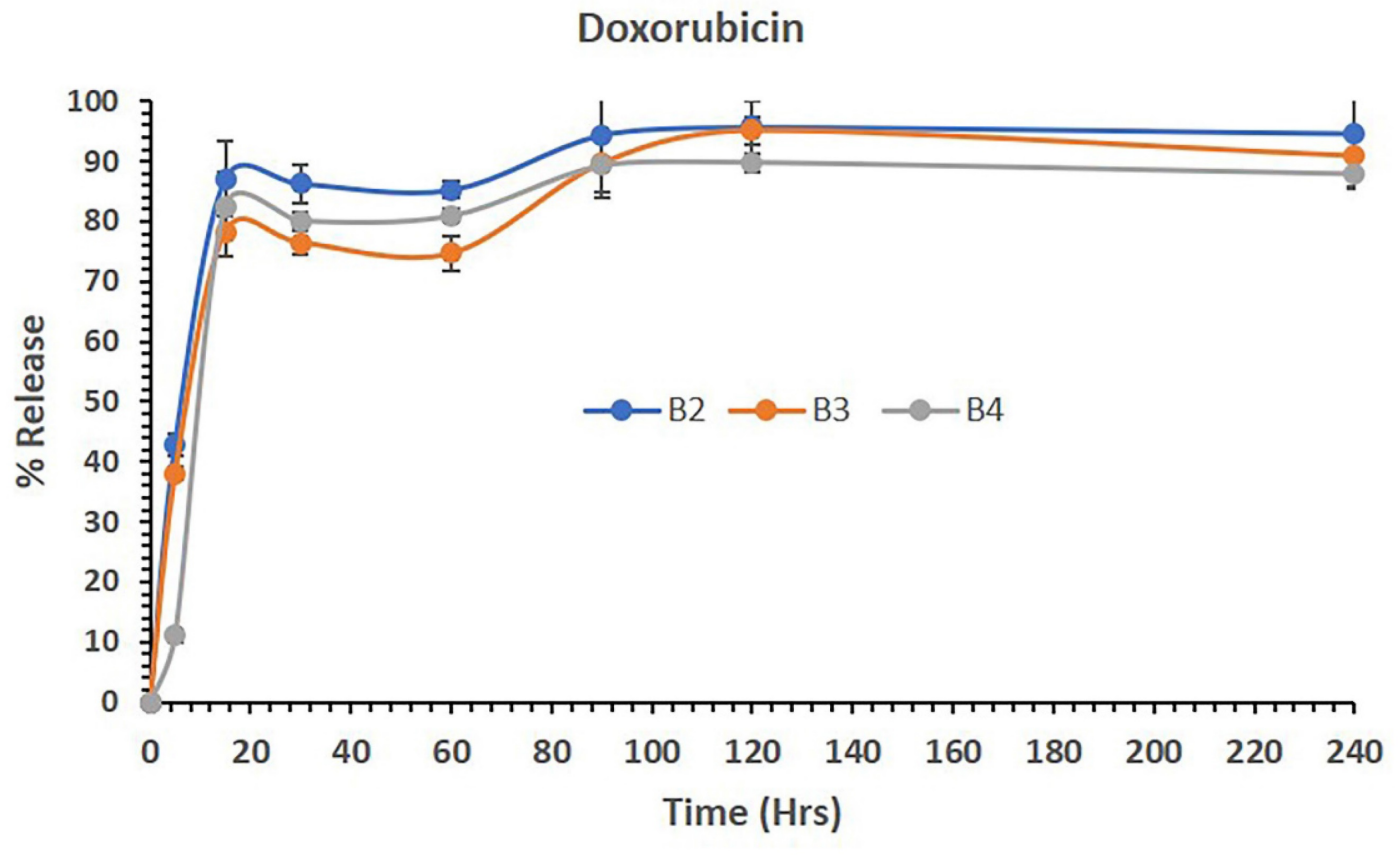
The percent release of DOX from the polymeric micelles of batches B2, B3 and B4 in aqueous media over 240 mins. The data are presented as the mean ±SD (n=3).

Figure 5 shows the release of DOX from polymeric micelles of batches B2, B3 and B4 in aqueous media. The data showed that DOX-loaded polymeric micelles (B2) released 42,94% of the drug in 5 mins and continuously released up to 94%, thus maintaining almost all DOX in solution until 4 hrs (240 mins). Similarly, the combination of LUT and DOX loaded in B3 and B4 PM batches released approximately 38% and 11% of LUT in 5 mins, respectively. Although the initial release rates were low, LUT and DOX release were steady from the B2-B4 PMs. The combined dosage forms B3 and B4 continued to be released up to almost 90% and maintained the drug in solution until 4 hrs. The overall release studies suggest that DOX was released from the combined dosage forms effectively without precipitating and was stable as the combined delivery system with LUT for active therapy.

### 4.9. Measurement of % Cell Viability of DF-PMs, LUT-PMs/DOX-PMs and LUT-DOX-PMs

Here we evaluated the effects of different PMs loaded with LUT and/or DOX on the viability of cancer lines. We used two human cancer cell lines, such as breast cancer cell line (MCF7) and hepatocellular carcinoma cell line (HepG2), which represent different subtypes of cancer cells with different molecular characteristics and drug sensitivities. We also compared the PMs with free LUT and DOX, as well as with blank PMs (DF-PMs).

**Figure 6:**
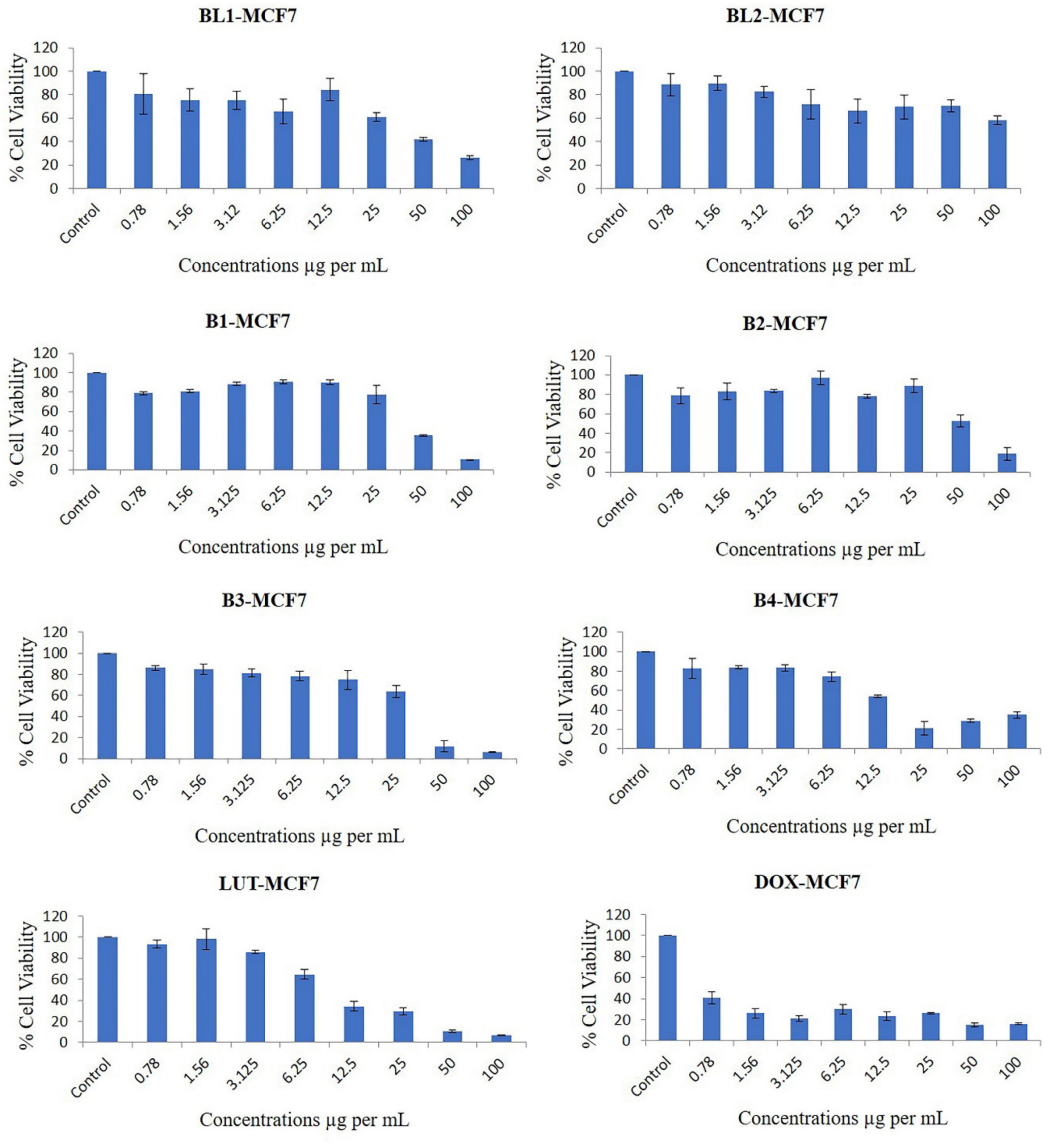
Effects of different concentrations of B1-LUT-PMs, B2 DOX-PMs, B3-LUT-DOX-PMs, B4- LUT-DOX-PMs, drug free BL1, drug free BL2 and pure drugs LUT & DOX on the % cell viability of breast cancer cell line MCF7 as measured by MTT 72 hrs following exposure.

Our results showed that all the PMs, except for BL2, inhibited the proliferation of breast cancer cells in a dose-dependent manner (Figure 6). The most potent PMs were B1, B2, B3, and B4, which contained both LUT and DOX in different ratios. These PMs reduced the cell viability by more than 50% at doses of 25 µg/mL or higher in both cell lines. These results suggest that the combination of LUT and DOX in PMs has a synergistic effect on the cytotoxicity of breast cancer cells, possibly by enhancing the intracellular delivery and accumulation of both drugs. Previous studies have reported that LUT and DOX can act synergistically to induce apoptosis, cell cycle arrest, and DNA damage in various cancer cells 32. Moreover, LUT can modulate the expression and activity of several drug resistance-related proteins, such as P-glycoprotein, multidrug resistance-associated protein 1, and breast cancer resistance protein, which can affect the efficacy of DOX 33, 34.

The PMs that contained only LUT (BL1) or only DOX (BD1) also inhibited the cell viability, but to a lesser extent than the combination PMs. BL1 was more effective than BD1 in both cell lines, indicating that LUT has a higher intrinsic cytotoxicity than DOX. This is consistent with previous reports that LUT has a broad spectrum of anticancer activities, such as inducing apoptosis, inhibiting angiogenesis, inflammation, and metastasis, and modulating various signalling pathways 35. DOX is a widely used chemotherapeutic agent for breast cancer, but its clinical use is limited by its cardiotoxicity and drug resistance 36. Therefore, LUT may be a promising alternative or adjuvant to DOX for breast cancer treatment.

BL2, which contained only DF-PMs, did not show any significant cytotoxicity in either cell line, even at the highest dose of 100 µg/mL. This indicates that the DF-PMs are biocompatible and non-toxic to normal cells, which is desirable for drug delivery systems. The DF-PMs are composed of diblock copolymers of poly(ethylene glycol) and poly(ε-caprolactone), which are known to be biodegradable and biocompatible. The DF-PMs can also improve the solubility and stability of LUT and DOX, which are both poorly soluble in water.

#### 4.9.1. The Original % viability for breast cancer cell line

The % of cell viability of B1 were 79.05, 80.91, 88.5, 90.36, 90.2, 77.66, 35.23 and 10.6 at doses of 0.78, 1.56, 3.12, 6.25, 12.5, 25, 50 and 100 µg/mL, respectively, compared with control (significance P values are Supplementary Table 1). The % of cell viability of B2 were 78.61, 83.04, 83.72, 96.97, 78.16, 88.8, 52.63 and 18.88 at doses of 0.78, 1.56, 3.12, 6.25, 12.5, 25, 50 and 100 µg/mL, respectively, compared with control.

The % cell viability of B3 were 86.12, 84.97, 81.34, 78.36, 74.77, 63.98, 11.75 and 6.27 at doses of 0.78, 1.56, 3.12, 6.25, 12.5, 25, 50 and 100 µg/mL, respectively, compared with control. The % cell viability of B4 were 82.94, 84.14, 83.44, 74.57, 54.2, 21.61, 29.14 and 34.9 at doses of 0.78, 1.56, 3.12, 6.25, 12.5, 25, 50 and 100 µg/mL, respectively, compared with control.

The % cell viability of BL1 were 80.81, 75.38, 75.15, 65.87, 84.37, 61.07, 41.85 and 25.97 at doses of 0.78, 1.56, 3.12, 6.25, 12.5, 25, 50 and 100 µg/mL, respectively, compared to control. The % cell viability of BL2 were 88.68, 89.81, 82.45, 71.97, 66.15, 69.54, 70.46 and 58.17 at doses of 0.78, 1.56, 3.12, 6.25, 12.5, 25, 50 and 100 µg/mL, respectively, compared with control.

The % cell viability of LUT were 93.3, 98.13, 85.6, 64.49, 34.26, 29.25, 10.74 and 6.63 at doses of 0.78, 1.56, 3.12, 6.25, 12.5, 25, 50 and 100 µg/mL, respectively, compared with control. The % cell viability of DOX were 40.63, 26.27, 21.35, 29.9, 23.31, 26.1, 15.33 and 16.16 at doses of 0.78, 1.56, 3.12, 6.25, 12.5, 25, 50 and 100 µg/mL, respectively, compared with control.

#### 4.9.2. The Original % viability for liver cancer cell line

The % of cell viability of B1 were 75.74, 71.55, 71.62, 74.94, 82.78, 99.34, 63.69 and 34.43 at doses of 0.78, 1.56, 3.12, 6.25, 12.5, 25, 50 and 100 µg/mL, respectively, compared with control (significance P values are supplementary Table 2). The % of cell viability of B2 were 69.01, 68.06, 70.89, 71.17, 81.91, 57.68, 35.18 and 21.12 at doses of 0.78, 1.56, 3.12, 6.25, 12.5, 25, 50 and 100 µg/mL, respectively, compared with control.

The % cell viability of B3 were 71.15, 63.24, 61.5, 56.13, 32.13, 20.52, 6.41 and 6.89 at doses of 0.78, 1.56, 3.12, 6.25, 12.5, 25, 50 and 100 µg/mL, respectively, compared with control. The % cell viability of B4 were 47.92, 46.74, 51.74, 44.07, 33.38, 22.79, 7.14 and 6.98 at doses of 0.78, 1.56, 3.12, 6.25, 12.5, 25, 50 and 100 µg/mL, respectively, compared with control.

The % cell viability of BL1 were 75.00, 71.39, 73.68, 73.5, 72.05, 75.69, 82.2 and 49.58 at doses of 0.78, 1.56, 3.12, 6.25, 12.5, 25, 50 and 100 µg/mL, respectively, compared to control. The % cell viability of BL2 were 77.83, 75.98, 70.73, 71.88, 68.99, 69.87, 82.4 and 78.59 at doses of 0.78, 1.56, 3.12, 6.25, 12.5, 25, 50 and 100 µg/mL, respectively, compared with control.

The % cell viability of LUT were 81.49, 77.64, 41.31, 27.56, 20.28, 14.82, 7.13 and 10.18 at doses of 0.78, 1.56, 3.12, 6.25, 12.5, 25, 50 and 100 µg/mL, respectively, compared with control. The % cell viability of DOX were 49.43, 38.54, 29.12, 28.45, 13.59, 7.53, 9.97 and 14.21 at doses of 0.78, 1.56, 3.12, 6.25, 12.5, 25, 50 and 100 µg/mL, respectively, compared with control.

**Figure 7:**
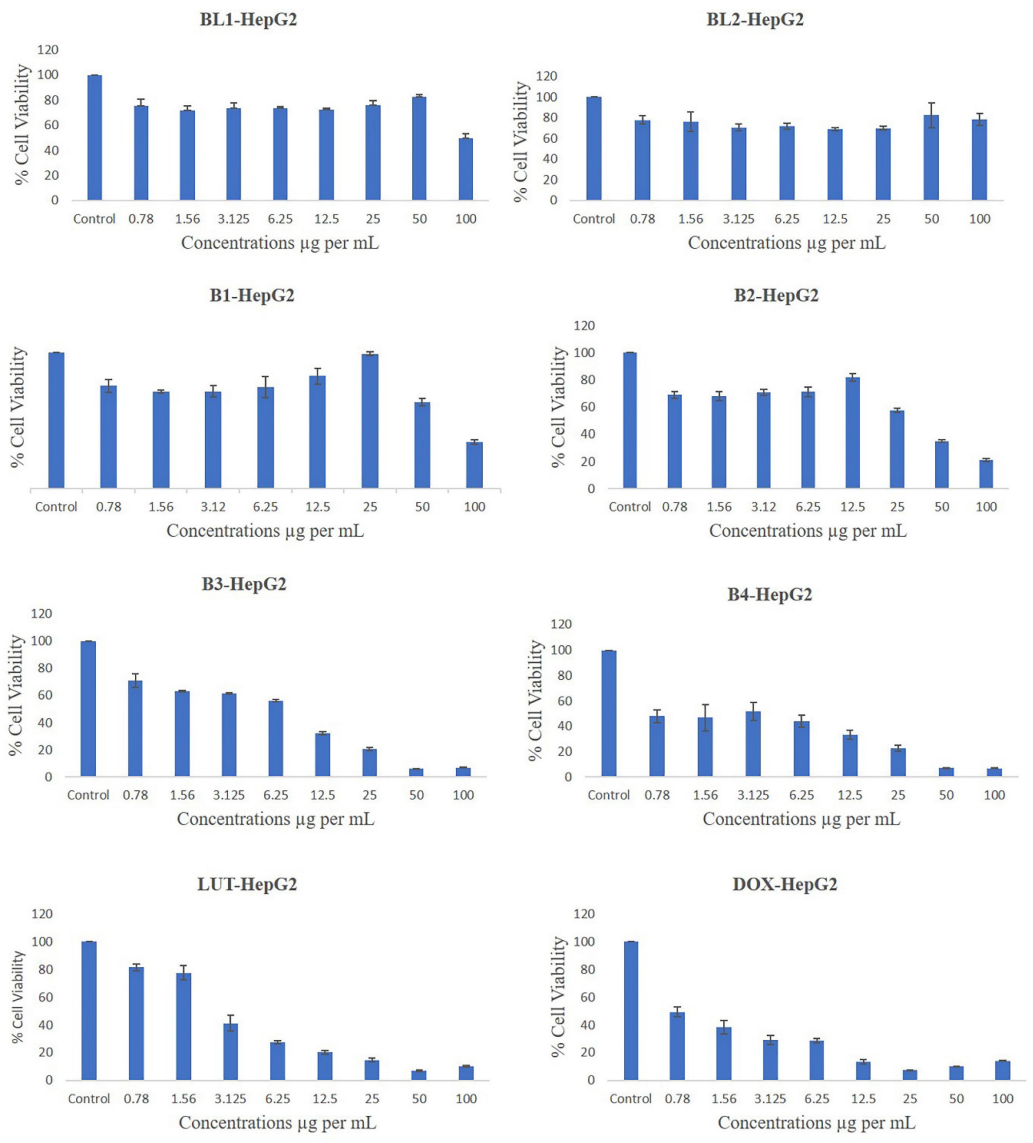
Effects of different concentrations of drug free BL1, drug free BL2, B1, B2, B3, B4, and pure drugs LUT & DOX on the % cell viability of liver cancer cell line (HepG2) as measured by MTT 72 hrs following exposure.

Figure 7 shows that the different PMs had varying effects on the cell viability of HepG2 cells, a human liver cancer cell line. HepG2 cells are commonly used as a model for studying liver metabolism, toxicity, and drug delivery. Among the PMs, BL1 and BL2 had the least inhibitory effect on HepG2 cells, with only the highest concentration of BL1 showing significant cytotoxicity. This suggests that these PMs are not effective carriers for anticancer drugs against HepG2 cells. On the other hand, B1, B2, B3, and B4 showed more potent anti-proliferative activity, with B3 and B4 being the most effective. These PMs may have enhanced the cellular uptake and intracellular release of the drugs, leading to increased apoptosis and cell cycle arrest of HepG2 cells. Previous studies have reported that PMs can improve the solubility, stability, and bioavailability of hydrophobic drugs, and can also target tumour cells by passive or active mechanisms 37.

LUT and DOX are two well-known anticancer drugs that have been widely used in clinical practice. The results showed that LUT and DOX alone could inhibit the proliferation of HepG2 cells in a dose-dependent manner, but the combination of LUT and DOX in PMs (LUT-DOX-PMs) had a synergistic effect, with the lowest IC50 value and the highest apoptotic rate among all the PMs. This indicates that LUT and DOX can act synergistically to induce more potent cytotoxicity and apoptosis in HepG2 cells. This may be due to the different mechanisms of action of LUT and DOX, which can target multiple pathways and overcome drug resistance 38. Moreover, using PMs as carriers may also increase the stability and delivery of LUT and DOX, and reduce their toxicity to normal cells and organs.

Concentrations causing 50% inhibition of growth of both cancer cells was used as IC50 value and it was calculated by trendline equation, as described earlier 39. The IC50 (inhibitory concentration at 50% viability) was quantified from the cell viability data of both cancer cell lines and as shown in Table 5. The IC50 was significantly lower in B3 and B4 than in B1 and B2 of both cancer cell lines. BL 1 showed a high IC50 value of 44.64±4.44 and 107.56±8.62 µg/mL, for breast and liver cancer cell line, respectively. On the other hand, BL 2 could not effectively inhibit 50% both cancer cells. Therefore, IC50 value could not be calculated for BL 2. LUT and DOX showed a lower IC50 value for both cancer cell lines (Table 5).

Breast cancer remains a significant health concern worldwide, with tumor invasion and metastasis being critical processes contributing to its morbidity and mortality. Understanding the mechanisms underlying these processes and identifying potential therapeutic targets is paramount in improving patient outcomes. Polymeric micelles (PMs), particularly those containing LUT and DOX, could emerge as promising candidates in this regard. Several studies 40 have elucidated the impact of PMs on breast cancer cell migration, highlighting their potential as therapeutic agents. Notably, PMs containing LUT have demonstrated efficacy in inhibiting the migration of breast cancer cells *in vitro* and *in vivo*. LUT, a flavonoid with known anti-cancer properties, exerts its effects through multiple mechanisms, including the downregulation of matrix metalloproteinases, crucial enzymes involved in extracellular matrix degradation.

Two different cancer cell lines such as breast (MCF-7) and liver (HepG2) cancer cells were used in this study and treated with different formulations to observe their anti-proliferation activity by MTT assay as mentioned in material and methods section. Cell proliferation is essential biological parameter for any living cells. Optical density was measured during MTT assay. Any increment or decline in optical density represent proliferation or anti-proliferation of any cells, respectively 41. In this study, we observed decline in optical density values in all formulations treated of both cancer cells compared to control, which indicates effectively treatment of cancer cells. Therefore, these formulations may be use for cancer treatment in future. However, further investigations are required to observe formulations' effects at gene and protein levels. Additionally, preclinical and clinical studies are warranted to evaluate the safety and efficacy of these PMs in breast cancer treatment, with the ultimate goal of improving patient outcomes and reducing disease burden.

## Conclusion

Our studies demonstrated that LUT-DOX-PMs with P407 and Gelucire 44/14 were effectively developed which induced significant reduction in cancer cell proliferation. These factors, when considered with its superior stability as shown by the zeta potential, improve the killing of breast cancer cells in *in vivo* systems although this remains to be extensively determined. The success of these studies may provide the groundwork for the future development of a superior pre-clinical high-throughput model. These findings might have potential application in cancer treatment in future.

## Supplementary Material

Supplementary tables.

## Figures and Tables

**Table 1:** Composition of the formulations studied in the experiments (ratio of mM for the chemicals expressed in %)

Formulation	P407	TPGS	GC	P188	PDLG	LUT	DOX
BL1	70%	30%	-	-	-	-	-
BL2	70%	-	30%	-	-	-	-
B1	74.3%	20.7%	-	-	-	5%	-
B2	70%	-	25%	-	-	-	5%
B3	74.3%	20.7%	-	-	-	2.5%	2.5%
B4	70%	-	25%	-	-	2.5%	2.5%
B5	9.45%	-	-	73.5%		12.6%	-
B6	31.9%	-	-	-	63.1%	5%	-

P407: Poloxamer 407; TPGS: Tocopheryl polyethylene glycol succinate; GC: Gellucire 44/14; P188: Poloxamer 188; PDLG: Poly(lactic-co-glycolic acid); LUT: Luteolin; Dox: Doxorubicin

**Table 2:** Measurement of the particle size, polydispersity index and zeta potential values of polymeric micelles before lyophilization

Formulation Batch	Particle size (nm)	PDI	Zeta Potential (mV)
BL1	24.2 ± 5.22	0.19 ± 0.042	NC
BL2	270.03 ± 4.38	0.74 ± 0.032	NC
B1	114.56 ± 4.03	0.21 ±0.013	-8.10 ±0.15
B2	121.36 ± 2.45	0.25 ±0.014	-5.81 ±0.25
B3	62.03 ±3.99	0.33 ±0.054	-2.27 ±0.11
B4	91.96 ± 5.80	0.59 ± 0.032	-7.78 ± 0.10
B5	2622 ±8.42	0.338 ±0.062	NC
B6	4159.333 ±7.84	0.83 ±0.048	NC

The data are presented as the mean **±** SD, (n= 3). NC# not calculated

**Table 3:** The drug loading and entrapment efficiency of the PMs

Formulation Batch	LUT (mg/g)	% Loading	DOX (mg/g)	%Loading
B1 (LUT-PMs)	31.80±0.39	3.18	X	X
B2 (DOX-PMs)	X	X	41.13±2.44	4.11
B3 (LUT-DOX-PMs)	64.92±1.71	6.49	19.16±0.26	1.92
B4 (LUT-DOX-PMs)	86.51±2.86	8.65	31.04±0.69	3.10

**Table 4:** Measurement of the particle size, polydispersity index and zeta potential values of polymeric micelles after lyophilization

Formulation Batch	Particle size (nm)	PDI	Zeta Potential (mV)
B1	414.96 ± 5.68	0.57 ±0.01	-7.23 ± 0.08
B2	455.23 ± 5.63	0.35 ± 0.03	-6.02 ± 0.58
B3	834.2 ± 7.25	0.34 ±0.24	-2.76 ± 0.51
B4	933.53 ±8.02	0.79 ±0.17	-7.98 ± 0.18

**Table 5:** IC_50_ values of different formulations of breast (MCF-7) and liver (HepG2) cancer cell lines

Name of formulation	MCF-7 (µg per mL)	HEPG2 (µg per mL)
B 1	40.91±1.99	77.41±1.06
B 2	62.1±4.41	36.04±0.44
B 3	28.94±2.25	7.27±0.21
B 4	14.89±1.1	3.39±0.98
BL 1	44.64±4.44	107.56±8.62
BL 2	NC#	NC#
LUT	9.44±0.26	3.32±0.7
DOX	2.16±0.09	2.59±0.16

NC# Not Calculated

## References

[1] Zheng S, Cheng Y, Teng Y, Liu X, Yu T, Wang Y (2017). Application of luteolin nanomicelles anti-glioma effect with improvement in vitro and in vivo. Oncotarget.

[2] Nabavi SF, Braidy N, Gortzi O, Sobarzo-Sanchez E, Daglia M, Skalicka-Woźniak K (2015). Luteolin as an anti-inflammatory and neuroprotective agent: A brief review. Brain Res Bull.

[3] Lin Y, Shi R, Wang X, Shen HM (2008). Luteolin, a flavonoid with potential for cancer prevention and therapy. Curr Cancer Drug Targets.

[4] Seelinger G, Merfort I, Schempp CM (2008). Anti-oxidant, anti-inflammatory and anti-allergic activities of luteolin. Planta Med.

[5] Majumdar D, Jung KH, Zhang H, Nannapaneni S, Wang X, Amin AR (2014). Luteolin nanoparticle in chemoprevention: in vitro and in vivo anticancer activity. Cancer Prev Res (Phila).

[6] Alshehri S, Imam SS, Altamimi MA, Hussain A, Shakeel F, Elzayat E (2020). Enhanced Dissolution of Luteolin by Solid Dispersion Prepared by Different Methods: Physicochemical Characterization and Antioxidant Activity. ACS Omega.

[7] Shakeel F, Haq N, Alshehri S, Ibrahim MA, Elzayat EM, Altamimi MA (2018). Solubility, thermodynamic properties and solute-solvent molecular interactions of luteolin in various pure solvents. Journal of Molecular Liquids.

[8] Wu G, Li J, Yue J, Zhang S, Yunusi K (2018). Liposome encapsulated luteolin showed enhanced antitumor efficacy to colorectal carcinoma. Mol Med Rep.

[9] Shinde P, Agraval H, Singh A, Yadav UCS, Kumar U (2019). Synthesis of luteolin loaded zein nanoparticles for targeted cancer therapy improving bioavailability and efficacy. Journal of Drug Delivery Science and Technology.

[10] Liu Z, Bi Y, Sun Y, Hao F, Lu J, Meng Q (2017). Pharmacokinetics of a liposomal formulation of doxorubicin in rats. Saudi Pharmaceutical Journal.

[11] Fritze A, Hens F, Kimpfler A, Schubert R, Peschka-Süss R (2006). Remote loading of doxorubicin into liposomes driven by a transmembrane phosphate gradient. Biochimica et Biophysica Acta (BBA) - Biomembranes.

[12] Patro NM, Devi K, Pai RS, Suresh S (2013). Evaluation of bioavailability, efficacy, and safety profile of doxorubicin-loaded solid lipid nanoparticles. Journal of Nanoparticle Research.

[13] Qi J, Yao P, He F, Yu C, Huang C (2010). Nanoparticles with dextran/chitosan shell and BSA/chitosan core—Doxorubicin loading and delivery. International Journal of Pharmaceutics.

[14] Cabanes A, Even-Chen S, Zimberoff J, Barenholz Y, Kedar E, Gabizon A (1999). Enhancement of antitumor activity of polyethylene glycol-coated liposomal doxorubicin with soluble and liposomal interleukin 2. Clin Cancer Res.

[15] Aryal S, Grailer JJ, Pilla S, Steeber DA, Gong S (2009). Doxorubicin conjugated gold nanoparticles as water-soluble and pH-responsive anticancer drug nanocarriers. Journal of Materials Chemistry.

[16] Ke W, Zhao Y, Huang R, Jiang C, Pei Y (2008). Enhanced oral bioavailability of doxorubicin in a dendrimer drug delivery system. J Pharm Sci.

[17] Zhao MX, Zhao M, Zeng EZ, Li Y, Li JM, Cao Q (2014). Enhanced anti-cancer efficacy to cancer cells by doxorubicin loaded water-soluble amino acid-modified β-cyclodextrin platinum complexes. J Inorg Biochem.

[18] Batrakova EV, Bronich TK, Vetro JA, Kabanov AV (2006). Polymer Micelles as Drug Carriers. Nanoparticulates as Drug Carriers.

[19] Jhaveri AM, Torchilin VP (2014). Multifunctional polymeric micelles for delivery of drugs and siRNA. Front Pharmacol.

[20] Zhang Y, Huang Y, Li S (2014). Polymeric micelles: nanocarriers for cancer-targeted drug delivery. AAPS PharmSciTech.

[21] Lu Y, Park K (2013). Polymeric micelles and alternative nanonized delivery vehicles for poorly soluble drugs. Int J Pharm.

[22] Dumortier G, Grossiord JL, Agnely F, Chaumeil JC (2006). A review of poloxamer 407 pharmaceutical and pharmacological characteristics. Pharm Res.

[23] Sun C, Li W, Ma P, Li Y, Zhu Y, Zhang H (2020). Development of TPGS/F127/F68 mixed polymeric micelles: Enhanced oral bioavailability and hepatoprotection of syringic acid against carbon tetrachloride-induced hepatotoxicity. Food Chem Toxicol.

[24] Panigrahi KC, Patra CN, Jena GK, Ghose D, Jena J, Panda SK (2018). Gelucire: A versatile polymer for modified release drug delivery system. Future Journal of Pharmaceutical Sciences.

[25] Kazi M, F AN, Noman O, Alharbi A, Alqahtani MS, Alanazi FK (2020). Development, Characterization Optimization, and Assessment of Curcumin-Loaded Bioactive Self-Nanoemulsifying Formulations and Their Inhibitory Effects on Human Breast Cancer MCF-7 Cells. Pharmaceutics.

[26] Alshadidi A, Shahba AA-W, Sales I, Rashid MA, Kazi M (2021). Combined curcumin and lansoprazole-loaded bioactive solid self-nanoemulsifying drug delivery systems (Bio-SSNEDDS). Pharmaceutics.

[27] Hari SK, Gauba A, Shrivastava N, Tripathi RM, Jain SK, Pandey AK (2023). Polymeric micelles and cancer therapy: an ingenious multimodal tumor-targeted drug delivery system. Drug Deliv Transl Res.

[28] Patra A, Satpathy S, Naik PK, Kazi M, Hussain MD (2022). Folate receptor-targeted PLGA-PEG nanoparticles for enhancing the activity of genistein in ovarian cancer. Artificial Cells, Nanomedicine, and Biotechnology.

[29] H (2020). Shariare M, Afnan K, Iqbal F, A. Altamimi M, Ahamad SR, S. Aldughaim M, et al. Development and optimization of epigallocatechin-3-gallate (EGCG) nano phytosome using design of experiment (DoE) and their in vivo anti-inflammatory studies. Molecules.

[30] Tang SM, Deng XT, Zhou J, Li QP, Ge XX, Miao L (2020). Pharmacological basis and new insights of quercetin action in respect to its anti-cancer effects. Biomed Pharmacother.

[31] Yusuf A, Casey A (2020). Liposomal encapsulation of silver nanoparticles (AgNP) improved nanoparticle uptake and induced redox imbalance to activate caspase-dependent apoptosis. Apoptosis.

[32] Kciuk M, Gielecińska A, Mujwar S, Kołat D, Kałuzińska-Kołat Ż, Celik I (2023). Doxorubicin-An Agent with Multiple Mechanisms of Anticancer Activity. Cells.

[33] Prasher P, Sharma M, Singh SK, Gulati M, Chellappan DK, Zacconi F (2022). Luteolin: a flavonoid with a multifaceted anticancer potential. Cancer Cell Int.

[34] Rao PS, Satelli A, Moridani M, Jenkins M, Rao US (2012). Luteolin induces apoptosis in multidrug resistant cancer cells without affecting the drug transporter function: Involvement of cell line-specific apoptotic mechanisms. International Journal of Cancer.

[35] Farghadani R, Naidu R (2023). The anticancer mechanism of action of selected polyphenols in triple-negative breast cancer (TNBC). Biomed Pharmacother.

[36] Al-malky HS, Al Harthi SE, Osman A-MM (2019). Major obstacles to doxorubicin therapy: Cardiotoxicity and drug resistance. Journal of Oncology Pharmacy Practice.

[37] Ghosh B, Biswas S (2021). Polymeric micelles in cancer therapy: State of the art. J Control Release.

[38] Sabzichi M, Hamishehkar H, Ramezani F, Sharifi S, Tabasinezhad M, Pirouzpanah M (2014). Luteolin-loaded phytosomes sensitize human breast carcinoma MDA-MB 231 cells to doxorubicin by suppressing Nrf2 mediated signalling. Asian Pac J Cancer Prev.

[39] Kazi M, Alanazi Y, Kumar A, Shahba AA-W, Rizwan Ahamad S, Alghamdi KM (2023). Oral bioactive self-nanoemulsifying drug delivery systems of remdesivir and baricitinib: a paradigmatic case of drug repositioning for cancer management. Molecules.

[40] Kwon MJ (2023). Matrix metalloproteinases as therapeutic targets in breast cancer. Frontiers in Oncology.

[41] AlGhamdi KM, Kumar A, Ashour AE, AlGhamdi AA (2015). A comparative study of the effects of different low-level lasers on the proliferation, viability, and migration of human melanocytes in vitro. Lasers Med Sci.

