# Supplementary material for: Development of Polymeric Micelles for Combined Delivery of Luteolin and Doxorubicin for Cancer Therapy
Source: J Cancer. 2024 Jul 8;15(14):4717–30. doi: 10.7150/jca.96402 (PMC11242336; doi:10.7150/jca.96402)
Supplement: Supplementary file 1 — Supplementary tables. [file jcav15p4717s1.pdf]

Development of Polymeric Micelles for Combined Delivery of Luteolin and Doxorubicin for Cancer Therapy

Supplementary data

The statistical analysis and results.

Table 1: Significance values obtained from one-way ANOVA assessment of the means of three cell culture experiments examining the cytotoxicity of human breast cancer cell line treated with different drugs.

Concentrations of drugs (µg per mL	p-value of drugs on human breast cancer cells (MCF-7)							
	BL1	BL2	B1	B2	B3	B4	LUT	DOX
Control vs 0.78	No	No	P < 0.0001	P < 0.001	P < 0.01	P < 0.001	No	P < 0.0001
Control vs 1.56	No	No	P < 0.0001	P < 0.01	P < 0.001	P < 0.001	No	P < 0.0001
Control vs 3.12	P < 0.01	No	P < 0.01	P < 0.01	P < 0.0001	P < 0.001	P < 0.01	P < 0.0001
Control vs 6.25	P < 0.001	P < 0.001	No	No	P < 0.0001	P < 0.0001	P < 0.0001	P < 0.0001
Control vs 12.5	No	P < 0.001	No	P < 0.001	P < 0.0001	P < 0.0001	P < 0.0001	P < 0.0001
Control vs 25	P < 0.0001	P < 0.001	P < 0.0001	No	P < 0.0001	P < 0.0001	P < 0.0001	P < 0.0001
Control vs 50	P < 0.0001	P < 0.001	P < 0.0001	P < 0.0001	P < 0.0001	P < 0.0001	P < 0.0001	P < 0.0001
Control vs 100	P < 0.0001	P < 0.0001	P < 0.0001	P < 0.0001	P < 0.0001	P < 0.0001	P < 0.0001	P < 0.0001

The statistical data were obtained for human breast cancer cell line with different drugs with their different concentrations and for controls (no drug) using one-way ANOVA and the post hoc Tukey HSD test.

Table 2: Significance values obtained from one-way ANOVA assessment of the means of three cell culture experiments examining the cytotoxicity of human hepatocellular carcinoma cell line treated with different drugs.

Concentrations of drugs (µg per mL	p-value of drugs on human hepatocellular carcinoma cell line (HepG2)							
	BL1	BL2	B1	B2	B3	B4	LUT	DOX
Control vs 0.78	P < 0.0001	P < 0.001	P < 0.0001	P < 0.0001	P < 0.0001	P < 0.0001	P < 0.0001	P < 0.0001
Control vs 1.56	P < 0.0001	P < 0.0001	P < 0.0001	P < 0.0001	P < 0.0001	P < 0.0001	P < 0.0001	P < 0.0001
Control vs 3.12	P < 0.0001	P < 0.0001	P < 0.0001	P < 0.0001	P < 0.0001	P < 0.0001	P < 0.0001	P < 0.0001
Control vs 6.25	P < 0.0001	P < 0.0001	P < 0.0001	P < 0.0001	P < 0.0001	P < 0.0001	P < 0.0001	P < 0.0001
Control vs 12.5	P < 0.0001	P < 0.0001	P < 0.0001	P < 0.0001	P < 0.0001	P < 0.0001	P < 0.0001	P < 0.0001
Control vs 25	P < 0.0001	P < 0.0001	No	P < 0.0001	P < 0.0001	P < 0.0001	P < 0.0001	P < 0.0001
Control vs 50	P < 0.0001	P < 0.01	P < 0.0001	P < 0.0001	P < 0.0001	P < 0.0001	P < 0.0001	P < 0.0001
Control vs 100	P < 0.0001	P < 0.001	P < 0.0001	P < 0.0001	P < 0.0001	P < 0.0001	P < 0.0001	P < 0.0001

The statistical data were obtained for human hepatocellular carcinoma cell line with different drugs with their different concentrations and for controls (no drug) using one-way ANOVA and the post hoc Tukey HSD test.
